# Maternal Hyperhomocysteinemia Produces Memory Deficits Associated with Impairment of Long-Term Synaptic Plasticity in Young Rats

**DOI:** 10.3390/cells12010058

**Published:** 2022-12-23

**Authors:** Tatyana Y. Postnikova, Dmitry V. Amakhin, Alina M. Trofimova, Natalia L. Tumanova, Nadezhda M. Dubrovskaya, Daria S. Kalinina, Anna A. Kovalenko, Anastasiia D. Shcherbitskaia, Dmitry S. Vasilev, Aleksey V. Zaitsev

**Affiliations:** 1 Sechenov Institute of Evolutionary Physiology and Biochemistry of the Russian Academy of Sciences, St. Petersburg 194223, Russia; 2 Institute of Translational Biomedicine, St. Petersburg State University, St. Petersburg 199034, Russia; 3 Sirius University of Science and Technology, Sochi 354340, Russia; 4 D.O. Ott Research Institute of Obstetrics, Gynecology and Reproductive Medicine, St. Petersburg 199034, Russia

**Keywords:** homocysteine, long-term potentiation, NMDA receptor, hippocampus

## Abstract

Maternal hyperhomocysteinemia (HCY) is a common pregnancy complication caused by high levels of the homocysteine in maternal and fetal blood, which leads to the alterations of the cognitive functions, including learning and memory. In the present study, we investigated the mechanisms of these alterations in a rat model of maternal HCY. The behavioral tests confirmed the memory impairments in young and adult rats following the prenatal HCY exposure. Field potential recordings in hippocampal slices demonstrated that the long-term potentiation (LTP) was significantly reduced in HCY rats. The whole-cell patch-clamp recordings in hippocampal slices demonstrated that the magnitude of NMDA receptor-mediated currents did not change while their desensitization decreased in HCY rats. No significant alterations of glutamate receptor subunit expression except GluN1 were detected in the hippocampus of HCY rats using the quantitative real-time PCR and Western blot methods. The immunofluorescence microscopy revealed that the number of synaptopodin-positive spines is reduced, while the analysis of the ultrastructure of hippocampus using the electron microscopy revealed the indications of delayed hippocampal maturation in young HCY rats. Thus, the obtained results suggest that maternal HCY disturbs the maturation of hippocampus during the first month of life, which disrupts LTP formation and causes memory impairments.

## 1. Introduction

During prenatal period, the developing brain is very vulnerable. Adverse external influences and stresses can significantly disrupt normal brain development and result in impaired cognitive functions such as learning and memory, among many others [1,2,3,4]. 

Maternal hyperhomocysteinemia (HCY) is a common complication of pregnancy caused by high levels of the amino acid homocysteine in both maternal and fetal blood. HCY is known to cause structural abnormalities in the placenta, including blood circulation disorders, disturbing transport of nutrients and neurotrophic factors from mother to fetus [5]. Severe HCY can lead to abnormal neural tube formation and fetal death [6]. Mild HCY causes many physiological abnormalities in the offspring, including impaired synaptic activity due to an imbalance of excitation and inhibition in neural network [7,8] and cognitive impairment [9,10]. HCY may lead to neuronal death and neuroinflammation in the cortical areas and hippocampus [11,12,13]. The projection pyramidal neurons in both cortical tissue and hippocampus are shown to be more vulnerable to HCY [12,13]. 

A common consequence of maternal HCY is impaired cognitive ability and memory [9,10,14,15], but the data about their possible mechanisms are rather scarce. The hippocampus plays an important role in various cognitive functions, in particular, it is involved in memory consolidation, spatial memory and learning [16]. In terms of cellular mechanisms, it is long-term synaptic plasticity, including long-term potentiation (LTP) and long-term depression (LTD), that underlies learning and memory [17,18]. Since the hippocampus is very sensitive to stress [19], we hypothesized that it may be the hippocampal abnormalities that underlie the cognitive decline in HCY.

Therefore, in this work, using a maternal HCY model in rats [12], we investigated how maternal HCY affects hippocampal LTP in offspring and their cognitive function at the age of 20 days (P20–young) and 90 days (P90–adults). We also investigated the molecular and synaptic mechanisms of LTP impairments in young rats. 

## 2. Materials and Methods

### 2.1. Animals

Adult female Wistar rats (*n* = 36, weight 210–320 g) were obtained from the Rappolovo Animal Center (St. Petersburg, Russia). The animals were housed with a 12-h light and dark cycle and had free access to a 20% (*w/w*) protein commercial chow and clean drinking water throughout the study. Exclusion criteria included signs of illness and behavioral defects at the start of the study. All experimental protocols were performed in accordance with guidelines of the Declaration of Helsinki and approved by the Sechenov Institute of Evolutionary Physiology and Biochemistry RAS (protocol code 3/2020, 18 March 2020).

### 2.2. Model of Maternal HCY

To confirm pregnancy, the presence of sperm in vaginal swabs after mating of female rats was checked. The animals were then daily administrated with 0.15% aqueous L-methionine solution (0.6 g/kg of body weight) *per os* from the 4th day of pregnancy (E4) until delivery [12,20]. Chronic administration of methionine at this dose caused an increase in homocysteine levels also in the blood and brain of fetuses, as we showed previously [20] and is similar to homocysteine concentration found in the serum of patients with mild HCY [21]. Control animals received water. Female rats gave birth naturally. In a further study, their male offspring were used at P20 and P90 (Table 1). The day of birth was defined as P1.

Only males were included in this study, since the results of females in behavioral tests may vary depending on the phase of the estrous cycle [22].

### 2.3. Electrophysology

#### 2.3.1. Brain Slice Preparation

Rats were decapitated, their brains were quickly removed and placed in oxygenated (95% O_2_/5% CO_2_; 0 °C) artificial cerebrospinal fluid (ACSF) of the following composition: 126 mM NaCl, 24 mM NaHCO_3_, 2.5 mM KCl, 2 mM CaCl_2_, 1.25 mM NaH_2_PO_4_, 1 mM MgSO_4_, and 10 mM dextrose; pH = 7.3–7.4. Horizontal 350 μm slices were prepared using a vibratome HM 650 V (Microm International, Walldorf, Germany) as previously explained [23]. Next, the brain slices were incubated at 35 °C for 1 h.

#### 2.3.2. Field Excitatory Postsynaptic Potential (fEPSP) Recordings

All experiments were performed at 30 °C; fEPSPs were recorded with a glass microelectrode (0.5–1.0 MΩ) filled with ACSF from stratum radiatum in the CA1 hippocampal area using the Model 1800 Microelectrode AC Amplifier (A-M Systems, Carlsborg, WA, USA). Schaffer’s collaterals were stimulated with a bipolar nichrome electrode using an A365 stimulus isolator (WPI, Sarasota, FL, USA). Responses were digitized with NI USB-6211 (National Instruments, Austin, TX, USA). WinWCP v5 software (University of Strathclyde, Glasgow, UK) was used in all experiments.

The theta-burst stimulation (TBS) protocol was applied for LTP induction [24]. The LTP amplitude was determined as a ratio of fEPSP rising phase slopes assessed 50–60 min after the TBS and before it. The analysis of the recordings was performed offline using Clampfit 10.2 software (Axon Instruments, Sunnyvale, CA, USA).

A competitive antagonist of N-methyl-D-aspartate receptors (NMDAR) D,L-2-amino-5-phosphonovalerate (AP-5, 50 µM; Sigma-Aldrich, St. Louis, MO, USA) was used to investigate the involvement of these receptors in synaptic plasticity.

#### 2.3.3. Whole Cell Patch-Clamp Recordings

A Nikon FN-1 microscope (Nikon, Tokyo, Japan) with a differential interference contrast optics and a video camera (Grasshopper 3 GS3-U3-23S6M-C; FLIR Integrated Imaging Solutions Inc., Wilsonville, OR, USA) was used to visualize the pyramidal neurons of CA1 hippocampus. Borosilicate glass pipettes (3–5 MΩ) were manufactured using a P-1000 pipette puller (Sutter Instrument, Novato, CA, USA). The pipette solution had the following composition: Cs-methanesulfonate—127 mM, HEPES—10 mM, NaCl—10 mM, EGTA—5 mM, QX314—6 mM, ATP-Mg—4 mM, and GTP—0.3 mM with pH adjusted to 7.25 by adding CsOH. The EPC 10 USB Double (HEKA Elektronik GmbH, Reutlingen, Germany) patch-clamp amplifier controlled with the HEKA Patchmaster software was used for the whole-cell patch-clamp recordings. The access resistance was typically 10–20 MΩ during the experiment.

The type of and the positioning of the stimulating electrode were the same as for the field potential recording. NMDAR-mediated currents were isolated by adding gabazine (10 µM, Alamone Laboratories, Jerusalem, Israel) and DNQX (10 µM, Tocris Bioscience, Bristol, UK) to the recording solution. The NMDAR-mediated eEPSCs were induced by the TBS protocol with the stimulus current strength set to 300–500 µA and recorded at −30 mV. The data were lowpass-filtered at 5 kHz and digitized at 40 kHz.

### 2.4. Reverse Transcription Followed by Quantitative Real-Time PCR (RT-qPCR)

HCY rats were decapitated at 20 (P20) and 90 (P90) days of life. According to the rat brain atlas [25], the dorsal hippocampus was isolated using freezing microtome OTF5000 (Bright Instruments, Luton, UK). Total RNA was extracted with the ExtractRNA reagent (Evrogen, Moscow, Russia). RQ1 DNAase (Promega, Madison, WI, USA) treatment of the samples was carried out. The reverse transcription was performed using 1 μg of total RNA, 0.5 µg oligo-dT primers, 0.25 µg 9-mer random primers (DNA Synthesis Ltd., Moscow, Russia), and M-MLV reverse transcriptase (100 units per 1 µg of RNA; Evrogen, Moscow, Russia). Before the PCR step, all cDNA samples were diluted 10-fold.

PCR reactions were carried out in a total volume of 10 µL with 0.8 µL of cDNA, 0.75 units of TaqM-polymerase (Alkor Bio, St. Petersburg, Russia), 3.5 mM of Mg^2+^, specific forward and reverse primers, and TaqMan probes (see Appendix A Table A1). Multiplexes were used to conduct qPCR: *Grin1* (encoding GluN1) + *Grin2a* (encoding GluN2a), *Grin2b* (encoding GluN2b) + *Gria1* (encoding GluA1) + *Gria2* (encoding GluA2), and three reference genes multiplexes as described elsewhere [26]. PCR reactions were performed in a CFX96 Touch Real-Time PCR Detection System (BioRad, Hercules, CA, USA).

The expression of the genes studied was normalized to the geometric mean of *Gapdh*, *Hprt1*, *Ywhaz* (P20) and *Gapdh*, *Hprt1*, *Pgk1* (P90). RefFinder online tool (https://www.heartcure.com.au/reffinder/ accessed on 29 November 2022) was used to select reference genes. The relative mRNA level was calculated using the 2^−ΔΔCt^ method [27].

### 2.5. Western Blot

Western blot analysis was performed as previously described [28]. Briefly, hippocampal tissue was homogenized. The amount of total protein in the hippocampus tissue lysate was measured according to the Bradford protocol [29]. After electrophoresis, proteins were transferred from the gel to a polyvinylidene difluoride membrane. Primary antibodies anti-GluN1 (1:1000; rabbit polyclonal ab17345, Abcam, Cambridge, UK) and anti-actin (1:5000; rabbit polyclonal A5060, Sigma-Aldrich, St. Louis, MO, USA) were used. Immunoreactivity was detected with secondary goat anti-rabbit IgG (1:5000; ab6721, Abcam) bound to horseradish peroxidase. Bands were visualized by chemiluminescence method using the Optiblot ECL Ultra Detect Kit (Abcam, ab133409). The relative band intensities were measured by densitometry using Image Lab software (Bio-Rad Laboratories Inc., Hercules, CA, USA). For each sample, the ratio of GluN1 to actin band intensity was calculated.

### 2.6. Immunohistochemistry

The distributions of synaptopodin and PSD95 were analyzed on P20 and P90; nine animals were in control and HCY groups. After transcardial perfusion by 4% paraformaldehyde in 0.1 M phosphate-buffered saline (PBS, pH 7.4), rats were decapitated, and the tissue blocks of their brains were immersed in the same fixative for 24 h, cryoprotected in 20% sucrose in PBS, frozen, and then sectioned at the coronal plane with a cryomicrotom (CM1510S, Leica Microsystem, Vetzlar, Germany). The 15-µm-thick sections were incubated first in the blocking serum (phosphate-buffered saline with 2% bovine serum albumin and 0.2% Triton X-100, pH 7.4) for 1 h at room temperature. Next, sections were incubated with anti-synaptopodin polyclonal antibody produced in rabbit (1:500, S9567, Sigma-Aldrich) and anti-PSD95 monoclonal antibody produced in mouse (1:400; ab13552, Abcam) in in phosphate-buffered saline with 1% bovine serum albumin for 24 h at 4 °C. FITC-conjugated antibodies against rabbit IgG (1:200, ab6717, Abcam) and Phycoerythrin (PE)-conjugated against mouse IgG (1:200, ab97024, Abcam) were used for the visualization.

The investigation was performed using a Leica SP5 confocal microscope (Leica Microsystem, Vetzlar, Germany). Ten sections per animal were examined (one 100 × 100 µm area per section). Actin-associated protein synaptopodin is localized in the in the dendritic spine neck of principal neurons [30,31]. The number of synaptopodin-positive clusters in CA1 of the dorsal hippocampus was counted using Master Morphology 4.2 software (VideoTest, Saint Petersburg, Russia).

### 2.7. Electron Microscopy

To investigate the ultrastructure of the CA1 of the dorsal hippocampus we analyzed three animals in each group at P20 and P90. After transcardial perfusion (1% of glutaraldehyde, 1% paraformaldehyde in 0.1 M PBS, pH 7.4), brain tissue was fixed in 1% OsO_4_, stained with uranyl acetate, dehydrated, and embedded in Araldite by the protocol described previously [32]. Ultra-thin (50 nm) sections were made using Leica ultramicrotome (UC7 RT, Leica Microsystem) and analyzed using a transmission electron microscope (FEI Tecnai Spirit V2, FEI, Hillsboro, OR, USA). The structural features of hippocampus cells and neuropile were analyzed in the blocks of brain tissue, starting at the level 4.5 mm from Bregma [25].

### 2.8. Behavioral Tests

For the analysis of the hippocampus-related cognitive functions we used widely-used paradigms: the novel object recognition (NOR) test [33] and Morris water maze test [34,35].

#### 2.8.1. Novel Object Recognition Test

The non-spatial memory in rats at P20 and P90 was examined using the NOR test. The rat was first placed in a special box for 5 min to adapt in the absence of any specific behavioral stimuli so that it could freely explore the experimental arena. Young rats were placed in a 45 × 45 × 13 cm box and in adult rats was placed a 100 × 100 × 30 cm box. Two hours after the adaptation to the experimental arena, during the training session, the animal was presented with two novel objects (object 1 and object 2) and left to explore these objects for 5 min. The total exploration time for both objects during the training or test sessions did not differ between control and HCY rats. After the training session, the animal was returned to its home cage. For memory test, the rat was returned to the experimental arena after different retention intervals: 5 min, 60 min or 24 h. In these test sessions, the object 1 was familiar for rats because it was staying unchanged during further testing, but the object 2 was always changed for a novel object. For each animal, the time (T) spent in tactile or olfactory contact with each object was recorded. To measure the cognitive function, we used the preference index (PI), which is calculated as the percentage of time spent studying one of two objects (object 1 or 2) or the novel object (i.e., object 3, 4 or 5) in the test sessions to the total time spent exploring both objects (novel and familiar). [28,36]. So, a 50% value indicated an equivalent preference for novel and familiar objects, and a value other than 50% indicated a positive or negative preference for the object. All the objects were made of thick glass. In preliminary experiments, it was shown that rats had no preference for the objects used in the test. In the experiment, two sets of objects were used small and large size, which were comparable to the body size of young and adult rats, respectively. The objects and the experimental arena were wiped with a 50% ethanol after each presentation.

#### 2.8.2. Morris Water Maze Test

Morris water maze test were performed to assess learning and spatial memory in rats at P20 (control: *n* = 8; HCY: *n* = 11) and P90 (control: *n* = 8; HCY: *n* = 10). Tests were carried out at same time every day for 5 consecutive days. A cylindrical tank (diameter: 150 cm) was filled with water (≈24 °C), made opaque with milk. An escape platform (diameter: 10 cm) was placed at a fixed position under the water surface (approximately 2 cm) and 20 cm from the pool wall. The tank was virtually divided into four quadrants, platform and platform zone (approximately 10 cm area around the platform). Videorecord and subsequent tracking by Noldus EthoVision XT v.11.5 (Noldus Inc., Leesburg, VA, USA) were used. Rats were trained to escape onto the hidden platform during 4 training days. Four (for young rats) or five (for adult rats) trials were conducted each day, and on the last day the performance of the acquired skill was tested in the absence of the platform. The animals were kept on the platform before the next swim, as well as after the end of a series of swims for 30 s. If a rat failed to reach the platform within 120 s, it was guided onto it (where it was kept for 20 s), and the latency was recorded as 120 s. In the probe trial (day 5), the platform was removed, and the rats were allowed to search it for 120 s. The speed, total distance, frequency of platform crossings and time spent in the target quadrant, platform area and platform zone were recorded, and the time spent by the animal searching for the platform (escape latency) was also recorded. At the end of the experiment, the animals were removed from the platform, dried and returned to their home cage.

### 2.9. Statistical Analysis

Statistical analysis of the data was carried out in SPSS 17 (IBM, Armonk, NY, USA), OriginPro 8 (OriginLab Corporation, Northampton, MA, USA), and GraphPad Prism 8.0.1 (GraphPad Software, San Diego, CA, USA). We used the Shapiro–Wilk test to check the normality of the distribution and the Leven’s test to check equality of variance. In behavior study we used two-way ANOVA and one sample *t*-test. The electrophysiological data were processed using a Student’s unpaired *t*-test (for two groups) or one-way/mixed model ANOVA with either Tukey’s Honestly Significant Difference (HSD) or Dunnett’s *post hoc* tests. Western blot, qPCR, and immunohistochemistry data were analyzed using an unpaired two-tailed *t*-test or Mann-Whitney *U*-test.

The results are considered significant when *p* < 0.05. All data are expressed as the mean ± the standard error of the mean.

## 3. Results

Prenatal pathologies cause numerous disturbances of development [37,38], which may have long-lasting consequences [39]. First, we investigated the effects of maternal HCY on learning and memory using Morris water maze and NOR tests.

### 3.1. Maternal HCY Impair Non-Spatial Memory in NOR Test

**Figure 1:**
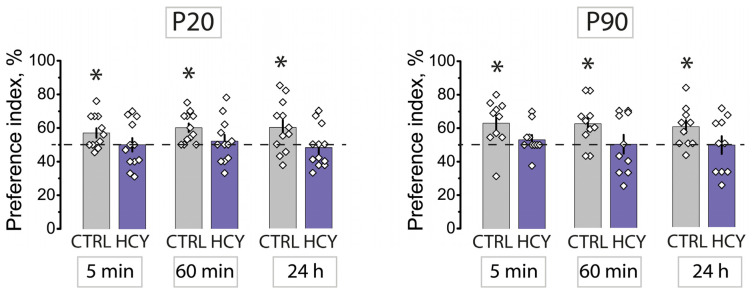
Diagrams showing preference index (PI) for novel objects in young (P20) and adult (P90) rats from the control (*n* = 22) and HCY (*n* = 22) groups. The horizontal dashed line marks a hypothetical 50% value indicating equivalent preference for new and familiar objects. All data in this and the following figures are presented as a mean ± standard error of the mean. * *p <* 0.05 (one-sample *t*-test, difference from 50% value).

NOR test was performed with control (*n* = 22) and HCY (*n* = 22) rats at P20 and P90. According to the paradigm of this test, a rat is considered to spend more time examining a new unfamiliar object compared to a known object. In the test series, one of the familiar objects was replaced with a new one, and PI was calculated for it. If the PI for the new subject was higher than 50%, we could assume that the rat memorized the old subject. Using a one-sample *t*-test, we calculated whether the PI was different from 50% in the different groups (Figure 1). In control animals, both young and adults, the PIs of new objects exceeded the 50% level, whereas in HCY rats the PI did not differ from 50% and thus HCY rats did not exhibit any preference for the novel objects. Thus, in young and adult HCY rats, non-spatial memory impairments were detected in the NOR test.

### 3.2. Maternal HCY Impair Learning and Spatial Memory in Morris Water Maze

**Figure 2:**
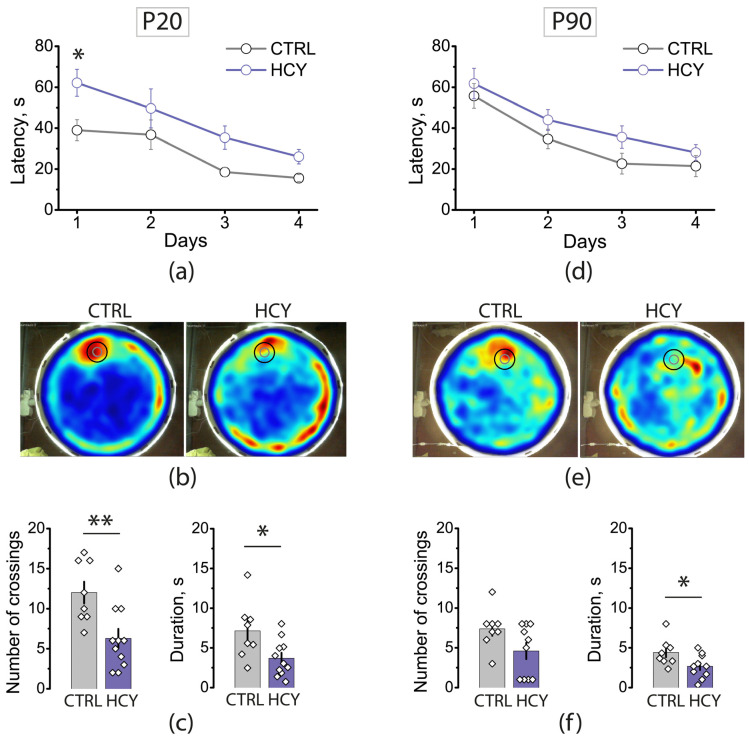
Morris water maze test in control and HCY rats performed at P20 (**a**–**c**) and P90 (**d**–**f**). (**a**,**d**) Average escape latency during training for 1–4 days. (**b**,**e**) Representative heat maps of Morris water maze obtained at test trial. The gray circle indicates the position of the rescue platform during training sessions, the black circle is the area that was used to count the number of crossings. Increasing color intensity (red) represents increased time spent. (**c**,**f**) The number of platform area crossings and time spent in the platform area by young (**c**) and adult (**f**) rats. * *p* < 0.05, ** *p* < 0.01; two-way ANOVA post hoc Bonferroni’s multiple comparisons for (**a**) and *t*-tests for (**c**,**f**).

Next, we evaluated the effects of prenatal HCY on learning and spatial memory in young and adult rats using MWM (Figure 2). An assessment of the learning ability can be done by measuring how the time to find the rescue platform changes during the four successive training days. Escape latency was averaged over 4 attempts in young rats and 5 attempts in adult rats. We found that prenatal HCY has a significant effect on learning in young (two-way ANOVA, F_1,68_ = 12.8, *p* < 0.001) and adult rats (F_1,64_ = 5.0, *p* = 0.03). However, significant differences in time to find the platform were revealed only in young rats on the 1st training day (control: 39 ± 5 s; HCY: 62 ± 7 s; *post hoc* Bonferroni’s multiple comparisons test, *p* < 0.05).

**Figure A1:**
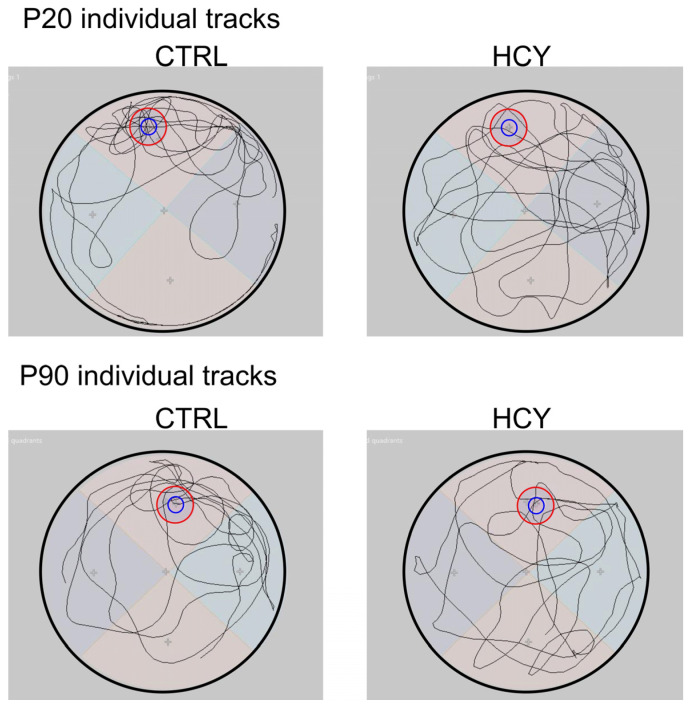
Representative examples of track in Morris water maze obtained at test trial. The blue circle indicates the position of the rescue platform during training sessions, the red circle is the area that was used to count the number of crossings.

Spatial memory was evaluated on day 5 (Appendix B Figure A1). On this day, the platform was removed from the pool, and we counted how many times the rat crossed the area where the rescue platform had been placed previously and how much time the rat spent in this area in total. Representative heat maps showed that young and adult control animals spent more time in the target area compared to HCY rats (Figure 2b,e). The mean number of platform area crossings was higher in young control rats compared to the HCY group (control: 12.0 ± 1.4; HCY: 6.3 ± 1.2; *t*-test, *p* < 0.01), whereas the number of crossings did not differ between adult control and HCY rats. However, time spent in the platform area was longer in control rats, both young (control: 7.1 ± 1.3 s; HCY: 3.7 ± 0.7 s; *t*-test *p* < 0.05) and adult (control: 4.4 ± 0.6 s; HCY: 2.7 ± 0.5 s; *t*-test *p* < 0.05).

Thus, maternal HCY has a negative effect on learning and spatial memory in offspring, with the impact evident even in adult animals.

### 3.3. Maternal HCY Impair Long-Term Synaptic Plasticity in the Rat Hippocampus

**Figure 3:**
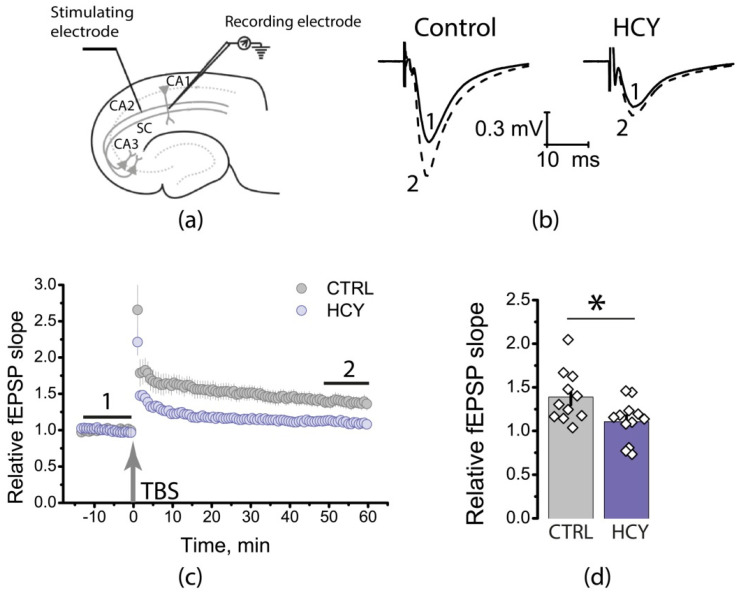
Long-term synaptic potentiation (LTP) is weakened in HCY rats. (**a**) Schema showing the positions of electrodes in the hippocampus. (**b**) Representative examples of fEPSP before induction (1) and 60 min after TBS (2). (**c**) Diagram showing changes in the value of the normalized slope of fEPSP in control (CTRL) and experimental (HCY) animals after theta-burst stimulation (TBS). (**d**) Diagram illustrates the differences in LTP between control (CTRL) and experimental (HCY) animals. * *p* < 0.05: a significant difference versus the control group (*t*-test).

The neural mechanism of memory is long-term synaptic plasticity. Because we identified impaired spatial memory, we investigated whether LTP in the CA3-CA1 hippocampal synapses of HCY rats is disturbed. We induced LTP with TBS protocol and found that LTP was significantly attenuated in the HCY group (control: 1.40 ± 0.09, *n* = 11; HCY: 1.11 ± 0.08, *n* = 12, *t*-test, *p* < 0.05; Figure 3).

**Figure 4:**
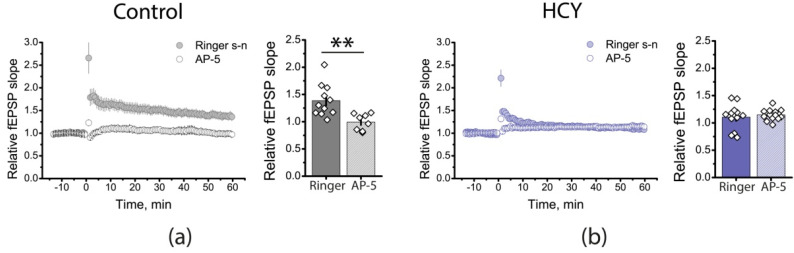
Effects of NMDARs blockade on LTP induction in the hippocampus of control and HCY rats. The normalized fEPSP slope in the presence of the NMDAR blocker AP-5 (50 μM), before and after TBS in the control (**a**) and experimental groups (**b**). ** *p* < 0.01: a significant difference (*t*-test).

LTP at CA3-CA1 synapses is believed to depend on NMDAR activation [40]. Since we observed an attenuation of LTP in HCY rats, it is possible that the NMDAR-dependent LTP mechanism is impaired. To test this assumption, we applied AP-5 (50 µM), a competitive antagonist of NMDARs, during the next experiments. In the control group, AP-5 application completely prevented LTP induction (Figure 4a; 0.99 ± 0.05; *n* = 8), these results confirm that LTP induction is an NMDAR-dependent process in control group.

In the HCY group, AP-5 does not affect the magnitude of LTP (Figure 4b; 1.15 ± 0.03; *n* = 13). However, because LTP is significantly reduced in the HCY group, no definite conclusion about the role of NMDARs in LTP can be drawn based on these experiments only.

### 3.4. Maternal HCY Affects the Properties of NMDAR-Mediated Currents in CA1

**Figure 5:**
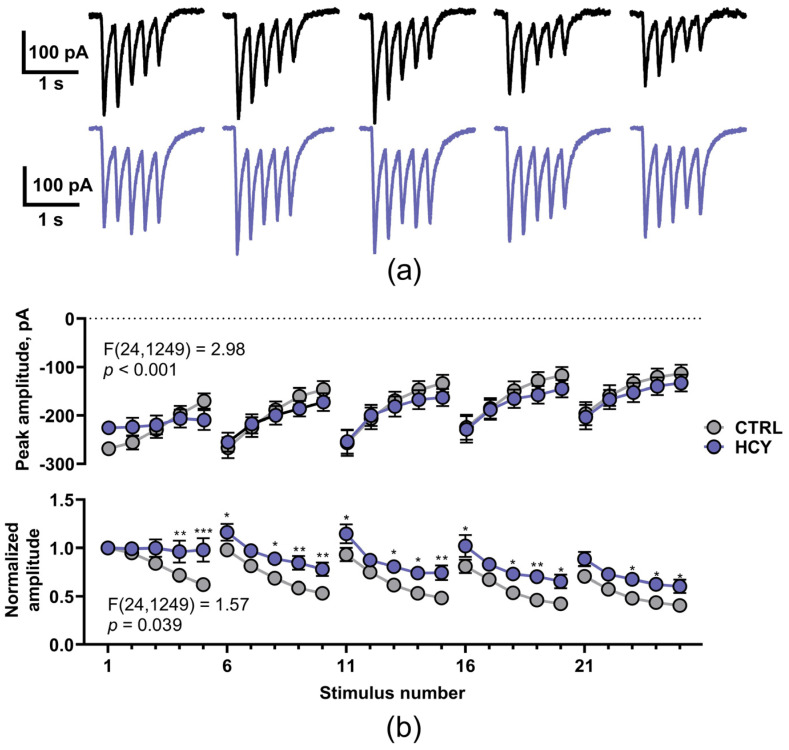
The altered properties of NMDAR-mediated currents evoked by TBS in HCY rats. (**a**) The representative voltage-clamp recordings of NMDAR-mediated currents, induced by TBS in CA1 neurons in control (black) and HCY rats (orange). (**b**) Upper trace: the average amplitudes of the individual peaks of current within the responses (*n* = 25 both for control and HCY groups); Lower trace: the same dataset, normalized to the amplitude of the first response. In both cases, the mixed-model ANOVA revealed a significant interaction between the factors (HCY × stimulus number; the *p*-values are reported on the figure). Asterisks indicate the significant difference between control and HCY rats (the values, which correspond to the same stimulus number, were compared using the Dunnett’s post hoc test).

The lack of LTP in HCY rats can be attributed to the weakening of the Ca^2+^ entry through NMDARs. Therefore, we compared the NMDAR-mediated response during TBS using the whole-cell patch-clamp technique. The NMDAR-mediated postsynaptic currents, evoked by TBS are presented at Figure 5a. In both control and HCY rats, the TBS protocol induced 5 current responses each consisting of 5 current peaks, yielding a total of 25 current peaks.

First, we compared the amplitude of current peaks in control and HCY rats using the mixed-model ANOVA; the analysis demonstrated that the effect of HCY depended on the peak number within the TBS (Figure 5b, upper trace), though the consequent Dunnett’s *post hoc* test did not detect any specific differences. So, next we compared the peak amplitudes, normalized to the value of the first current peak (Figure 5b, lower trace). The mixed-model ANOVA analysis also demonstrated the significant interaction between the factors, and the consequent *post hoc* test revealed that the HCY rats sustained a higher response amplitude to the end of each of 5 current bursts, compared to the control rats.

Thus, our results demonstrate that prenatal exposure to HCY does not reduce the magnitude of NMDAR-mediated currents, evoked by TBS in the hippocampal neurons of the offspring rats. However, it decreases the desensitization of NMDARs, which might change temporal calcium signals in synapses and lead to LTP impairment.

### 3.5. Effects of HCY on Expression of Ionotropic Glutamate Receptor Subunits

**Figure 6:**
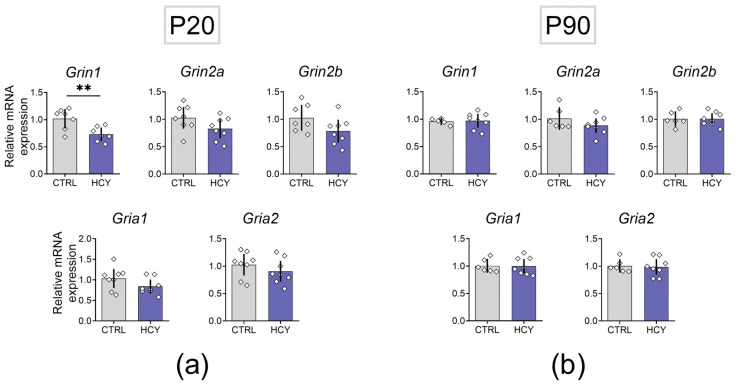
The relative mRNA expression of *Grin1*, *Grin2a*, *Grin2b* subunits of NMDA receptors and *Gria1*, *Gria2* subunits of AMPA receptors in the dorsal hippocampus of HCY rats at P20 (**a**) and P90 (**b**). CTRL—control group, HCY—experimental animals. Unpaired *t*-test, ** *p* < 0.01.

Impaired synaptic plasticity may be caused by changes in the expression of ionotropic glutamate receptor subunits. Therefore, we analyzed possible changes in the gene expression of *Grin1*, *Grin2a*, *Grin2b* subunits of NMDA receptors and *Gria1*, *Gria2* subunits of AMPA receptors the dorsal hippocampus of HCY rats at P20 and P90. We revealed that *Grin1* gene expression decreases at P20 (*t* = 3.2, *p* < 0.01). This result suggests a possible change in the total number of NMDA receptors as the GluN1 subunit encoded by the *Grin1* gene is obligate for this type receptors. However, at P90, the mRNA production of the *Grin1* gene returned to control values. No changes in the expression of other genes encoding different subunits of NMDA and AMPA receptors were detected. No differences between groups were observed in adult rats (Figure 6).

**Figure 7:**
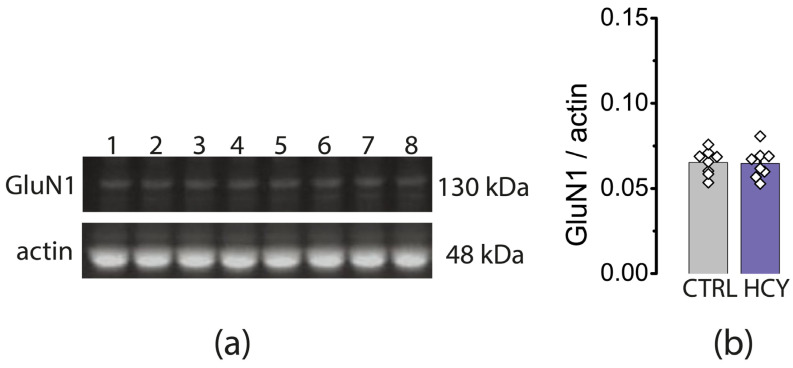
Western blot of GluN1 subunit of NMDAR in control (1,3,5,7) and HCY (2,4,6,8) rats on P20. On (**a**) the representative bands of the GluN1 and actin protein. (**b**) presents the results of densitometry of GluN1 band in CTRL—control group and HCY—experimental animals. Unpaired Mann- Whitney *U*-test *p* = 0.88.

Since the level of Grin1 mRNA in P20 HCY rats appeared to be decreased, the level of the GluN1 subunit of the NMDA receptor was additionally assayed by the Western blot method. We found that the protein level of the GluN1 subunit in the hippocampal tissue of HCY rats was the same as in controls (*U* = 30, *p* = 0.88, Figure 7). 

Thus, our experiments suggest that prenatal exposure to HCY does not cause significant changes in the expression of glutamate receptor subunits in the hippocampus, and it is not likely to cause impaired synaptic plasticity.

### 3.6. The Number of Synaptopodin-Positive Dendritic Spines Decreased in the Hippocampus Following Prenatal HCY

**Figure 8:**
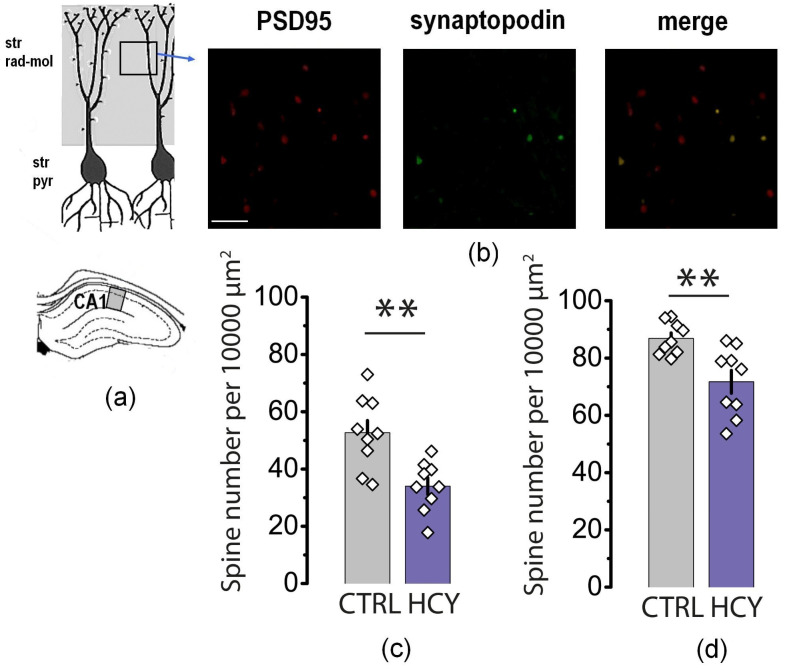
The distribution of actin-associated protein synaptopodin in the stratum radiatum-moleculare in CA1 of dorsal hippocampus. (**a**) Scheme showing the area of interest in *stratum radiatum-moleculare* of CA1. (**b**) Distribution of an actin-associated protein synaptopodin (green) and postsynaptic protein PSD95 (red) in the hippocampus of control rat at P20. Scale bar = 10 µm. (**c**,**d**) Diagrams showing the average number of synaptopodine-positive clusters in the hippocampus of P20 (**c**) and P90 (**d**) control and HCY rats. ** *p* < 0.01, unpaired two-tailed Mann-Whitney *U*-test.

Another possible mechanism of LTP decrease, which may contribute to memory and learning impairment, is a decline in the number of labile synaptopodin-positive dendritic spines. Synaptopodin is a specific marker of labile mushroom spines [41]. Previous studies have shown that these mushroom-shaped spines play a key role in neuronal plasticity [42,43,44]. Therefore, using the immunofluorescence method, we compared the number of synaptopodin-positive clusters in the CA1 region of the dorsal hippocampus in control Band HCY animals (Figure 8).

We first confirmed that synaptopodin was localized in postsynaptic density, for which slices were double stained for synaptopodin and the postsynaptic marker protein PSD95. The results showed that all synaptopodin clusters were colocalized with PSD95-positive spots (Figure 8b), confirming that synaptopodin-positive specks corresponded to dendritic spines.

In P20 HCY rats, the number of synaptopodin-positive dendritic spines was only 64 ± 6% of the control level (*U* = 8, *p* < 0.01, Figure 8c). In P90 HCY rats, the number of synaptopodin-positive dendritic spines was 83 ± 5% of the control level (*U* = 9, *p* < 0.01, Figure 8d). The obtained data strongly suggest that the number of synaptopodin-positive spines is reduced in the dorsal hippocampus of HCY rats.

### 3.7. Ultrastructure of the CA1 Hippocampus Tissue

**Figure 9:**
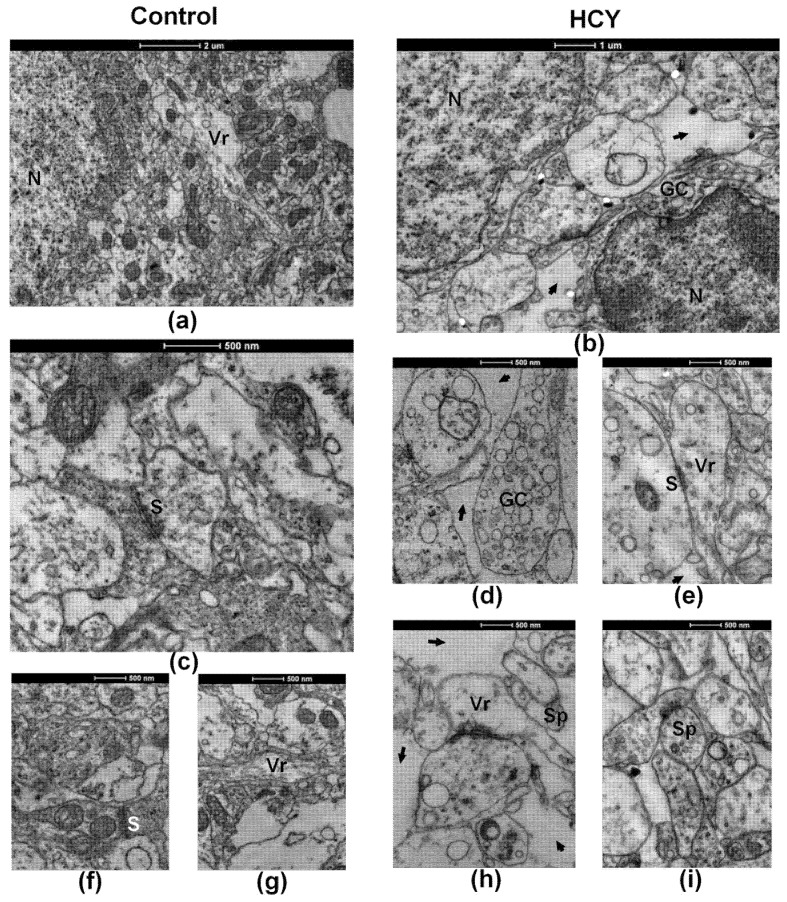
Electron microscope examination of the CA1 region of the dorsal hippocampus of control (**a**,**c**,**f**,**g**) and HCY (**b**,**d**,**e**,**h**,**i**) rats at P20. Sp—dendritic spines, S—synaptic contacts, Vr—varicose shafts, GC—growth cone, intercellular spaces are marked with arrows. Scale bars: 2 µm (**a**), 1 µm (**b**), 500 nm (**c**–**i**).

Next, we investigated the ultrastructure of hippocampus tissue with electron microscopy. On P20, we found signs indicating delayed hippocampal maturation in HCY rats as compared with control group; in particular, we found an increase in the volume of extracellular space (Figure 9b,h vs. Figure 9a,f) and the presence of many growth cones (Figure 9b,d vs. Figure 9a). Synaptic contacts were mostly symmetrical (Figure 9e,h vs. Figure 9c,f), there was usually a reduced number of synaptic vesicles in synaptic terminals (Figure 9h,i vs. Figure 9c), and dendritic spines were underdeveloped (Figure 9h,i).

At P20, both groups showed a small number of varicose extensions on neuronal processes (Figure 9a,g versus Figure 9e), but unlike control animals, in which varicose extensions form mature synaptic contacts with axon terminals, HCY rats had only a few immature synaptic contacts with underdeveloped postsynaptic densities and rare synaptic vesicles on most varicose extensions (Figure 9e,g). The revealed delay in maturation of the dorsal hippocampus of HCY rats may be responsible for the decrease in synaptic plasticity.

By 3 months of postnatal development in both control and HCY rats, all signs of CA1 immaturity of the dorsal hippocampus were completely abolished.

These data suggest that HCY leads to a delay in hippocampal maturation during the first month of life. Delayed synaptic development and decreased number of mature dendritic spines may be responsible for the decreased LTP found in this study.

## 4. Discussion

Prenatal development is the most vulnerable period for the brain formation. Any disturbance during this period can have a significant impact on cognitive functions at a later ontogenesis due to the disruption of neural networks [14,45,46].

In this study, we found that maternal HCY affects hippocampus-dependent learning and memory in the NOR and Morris water maze tests in the offspring which is consistent with previous studies [47,48]. The cognitive decline was observed in young (P20) and adult (P90) animals. In the NOR test, where the animal’s innate motivation to explore the environment is used to assess memory [49], the lack of preference for a new object indicated memory decline in HCY rats. Similar results were obtained using the Morris water maze test. We found that prenatal HCY has a significant effect on learning in young and adult rats, and it impairs their spatial memory.

Since the long-term synaptic plasticity is considered to be a cellular mechanism of memory, we investigated the properties of long-term synaptic plasticity in the CA1 region of the hippocampus. We revealed that LTP was significantly attenuated in the HCY group. Thus, impaired LTP in the hippocampal synapses may be the neurobiological mechanism of cognitive decline in HCY rats.

However, the cause of LTP attenuation in HCY rats is not yet fully understood. Since the mechanism of LTP production at CA3-CA1 synapses is NMDAR-dependent [34], we hypothesized that disruption of NMDAR signaling may be observed in HCY rats. Previously, it was shown in many models that disruption of the properties of NMDARs, changes in their subunit composition, can lead to weakening of long-term synaptic plasticity [23,24,28].

In addition, this hypothesis is supported by evidence that different prenatal stresses can change the subunit composition of ionotropic glutamate receptors. In the model of prenatal hypoxia-ischemia, *Grin1* gene expression was shown to decrease in the rat hippocampus at 4, 8 and 30 days of life [50], mRNA production of *Grin2a* and *Grin2b* did not change in Cai and Rhodes work [50]. The random variable prenatal stress led to increased mRNA levels of *Grin2b* in the hippocampus of Sprague-Dawley and Lewis rats [51]. Prenatal exposure to diesel exhaust particles causes a reduction hippocampal *Grin2a* expression in mice [52,53]. Furthermore, hippocampal *Gria1-4* expression was significantly decreased in prenatally stressed offspring rats [54].

It is known that homocysteine may induce calcium influx through NMDAR channel activation [55], Exposing primary cortical neuronal cultures to homocysteine leads to a sustained low-level increase in intracellular Ca^2+^ and consequent cell death [56] and therefore excessive activation of NMDARs in early ontogenesis can impair the development of the glutamatergic system. Therefore, we analyzed gene expression of *Grin1*, *Grin2a*, *Grin2b* subunits of NMDA receptors and *Gria1*, *Gria2* subunits of AMPA receptors in the dorsal hippocampus of HCY rats at P20 and P90. The expression of these receptors has not previously been studied in a model of maternal HCY. We found a decrease in mRNA production of GluN1 subunit but the expression of the other subunits of the NMDA and AMPA receptors was not altered.

The decrease in mRNA production of GluN1 subunit may indicate a reduction in the total number of NMDA receptors, as this subunit is obligatory [57]. To further verify this finding, we analyzed the protein level of the GluN1 subunit in the hippocampal tissue of HCY rats using Western blot, but these experiments failed to confirm differences between HCY and controls. One possible explanation for this mismatch could be the delay between transcription and translation under different pathological conditions [58,59]. Similar discrepancies between changes in gene expression and protein levels of NMDA receptor subunits have been detected in experimental models of neuroinflammation [60] and seizures [61].

Thus, our experiments show that maternal HCY does not cause significant changes in the expression of ionotropic glutamate receptor subunits in the hippocampus and, therefore, our hypothesis that impaired synaptic plasticity is due to changes in the expression level of glutamate receptors is not supported by experimental data.

However, even if the expression level of NMDA receptors and their subunit composition are not altered, their biophysical properties may be disturbed, leading to changes in the characteristics of calcium transients, which, in turn, may be the cause of LTP disorders. To test this assumption, we compared the NMDAR-mediated response during TBS using the whole-cell patch-clamp technique. We revealed that the magnitude of NMDA receptor-mediated currents did not change while their desensitization decreased in HCY rats. Multiple mechanisms can affect the desensitization of NMDARs, which include the modulation by intracellular signaling proteins and calcium ions [62,63,64]. Ambient zinc, as well as NMDAR coagonists glycine and serine also affect the desensitization kinetics of NMDARs [65,66]. Glycine-independent desensitization of NMDARs in vitro decreases during development due to the increase of the receptor synaptic localization [67]. Homocysteine reduces the desensitization of NMDARs in cultured neurons, which can be occluded by increasing the ambient glycine concentration [68], though it is very unlikely that high-enough levels of this amino acid persist during the postnatal development of rats in the implemented model. Since LTP induction is due to the activation of calcium-dependent molecular mechanisms, the slower kinetics of NMDARs desensitization indicates that these mechanisms may be impaired in HCY rats.

Another reason for the weakening of LTP may be the disturbance of functional maturation of synapses in HCY rats. Morphological data support this hypothesis. Electron microscopy revealed numerous signs of delayed synaptic maturation in the hippocampus of young HCY rats. In particular, HCY rats have a lower density of synapses with a mature spine apparatus that contain synaptopodin. The spine apparatus is an essential component of dendritic spines of cortical and hippocampal neurons and synaptopodin, an actin-binding protein, is tightly associated with the spine apparatus [41] and plays an important role in synaptic plasticity [69]. It was shown the dendritic spines of synaptopodin knockout mice lacked any spine apparatus. In these knockout animals a reduction in hippocampal LTP was also shown [41]. Overexpression of synaptopodin are crucial for the maintenance of the enlargement of dendritic spines in hippocampal neurons induced by the neuronal activity [31]. It was found that synaptopodin plays a crucial role in the calcium store-associated ability of neurons to undergo long-term plasticity of glutamate receptors [69]. A decrease in the number of spines containing synaptopodin should lead to a decrease in the LTP value, which is in perfect agreement with our experimental data.

Thus, our study shows that maternal HCY disturbs hippocampal maturation, in particular by reducing the number of synaptopodin-containing spines, which leads to attenuated LTP and impaired memory and learning in young rats. In addition, maternal HCY affects the properties of NMDAR-dependent currents by attenuating the desensitization of NMDARs. These changes can also affect plasticity. It should be noted that behavioral abnormalities persist in adult animals as well. Our experimental data convincingly demonstrate that maternal HCY is a substantial adverse factor for normal fetal development, affects brain maturation and cognitive ability in childhood and adulthood, and therefore requires immediate treatment.

## Figures and Tables

**Table 1:** The number of animals used in experiments.

Groups	Electro-Physiology	RT-qPCR	WB	IHC	EM	Behavioral Tests	
						NOR	MWM
P20 CTRL	13	7	8	9	3	12	8
P20 HCY	14	8	8	9	3	12	11
P90 CTRL	-	6	-	9	3	10	8
P90 HCY	-	8	-	9	3	10	10

RT-qPCR—Reverse Transcription Followed by Quantitative Real-Time PCR; WB—Western blot, IHC—Immunohistochemistry; EM—Electron microscopy; NOR—Novel object recognition test, MWM—Morris water maze test.

## Data Availability

The data presented in this study are available on request from the corresponding author.

## Appendix A

**Table A1:** Primers and probes (DNA Synthesis Ltd., Moscow, Russia) used for RT-qPCR.

Gene Symbol RefSeq Accession Number	Primer 5’–3’ (Forward, Reverse, Probe)	Final Concentrations, nM	References
*Actb* NM_031144	TGTCACCAACTGGGACGATA GGGGTGTTGAAGGTCTCAAA FAM-CGTGTGGCCCCTGAGGAGCAC-BHQ1	200 200	[70] (primers) [26] (probe)
*Gapdh* NM_017008	TGCACCACCAACTGCTTAG GGATGCAGGGATGATGTTC R6G-ATCACGCCACAGCTTTCCAGAGGG-BHQ2	200 100	[71]
*B2m* NM_012512	TGCCATTCAGAAAACTCCCC GAGGAAGTTGGGCTTCCCATT ROX-ATTCAAGTGTACTCTCGCCATCCACCG-BHQ1	200 100	[72]
*Rpl13a* NM_173340	GGATCCCTCCACCCTATGACA CTGGTACTTCCACCCGACCTC FAM-CTGCCCTCAAGGTTGTGCGGCT-BHQ1	200 100	[73] (primers) [26] (probe)
*Sdha* NM_130428	AGACGTTTGACAGGGGAATG TCATCAATCCGCACCTTGTA R6G-ACCTGGTGGAGACGCTGGAGCT-BHQ2	200 100	[74] (primers) [26] (probe)
*Ppia* NM_017101	AGGATTCATGTGCCAGGGTG CTCAGTCTTGGCAGTGCAGA ROX-CACGCCATAATGGCACTGGTGGCA-BHQ1	200 100	[75]
*Hprt1* NM_012583	TCCTCAGACCGCTTTTCCCGC TCATCATCACTAATCACGACGCTGG FAM-CCGACCGGTTCTGTCATGTCGACCCT-BHQ1	200 100	[76] (primers) [26] (probe)
*Pgk1* NM_053291	ATGCAAAGACTGGCCAAGCTAC AGCCACAGCCTCAGCATATTTC R6G-TGCTGGCTGGATGGGCTTGGA-BHQ2	200 100	[77] (primers) [26] (probe)
*Ywhaz* NM_013011	GATGAAGCCATTGCTGAACTTG GTCTCCTTGGGTATCCGATGTC ROX-TGAAGAGTCGTACAAAGACAGCACGC-BHQ1	200 100	[77] (primers) [26] (probe)
*Grin1* NM_017010	GTTCTTCCGCTCAGGCTTTG AGGGAAACGTTCTGCTTCCA FAM-CGGCATGCGCAAGGACAGCC-BHQ1	200 100	[78]
*Grin2a* NM_012573	GCTACACACCCTGCACCAATT CACCTGGTAACCTTCCTCAGTGA FAM-TGGTCAATGTGACTTGGGATGGCAA-BHQ1	200 100	[79]
*Grin2b* NM_012574	CCCAACATGCTCTCTCCCTTAA CAGCTAGTCGGCTCTCTTGGTT FAM-GACGCCAAACCTCTAGGCGGACAG-BHQ1	200 100	[79]
*Gria1* NM_031608	TCAGAACGCCTCAACGCC TGTAGTGGTACCCGATGCCA ROX-TCCTGGGCCAGATCGTGAAGCTAGAAAA-BHQ1	200 100	[80]
*Gria2* NM_017261	CAGTGCATTTCGGGTAGGGA TGCGAAACTGTTGGCTACCT FAM-TCGGAGTTCAGACTGACACCCCA-BHQ1	200 100	[80]
